# Review of Prodrug and Nanodelivery Strategies to Improve the Treatment of Colorectal Cancer with Fluoropyrimidine Drugs

**DOI:** 10.3390/pharmaceutics16060734

**Published:** 2024-05-29

**Authors:** Santu Sarkar, Sezgin Kiren, William H. Gmeiner

**Affiliations:** 1 Department of Cancer Biology, Wake Forest University School of Medicine, Winston-Salem, NC 27157, USA; sasarkar@wakehealth.edu; 2 Department of Chemistry, Winston-Salem State University, Winston-Salem, NC 27110, USA; kirens@wssu.edu

**Keywords:** fluoropyrimidine, prodrug, nanodelivery, colorectal cancer, thymidylate synthase

## Abstract

Fluoropyrimidine (FP) drugs are central components of combination chemotherapy regimens for the treatment of colorectal cancer (CRC). FP-based chemotherapy has improved survival outcomes over the last several decades with much of the therapeutic benefit derived from the optimization of dose and delivery. To provide further advances in therapeutic efficacy, next-generation prodrugs and nanodelivery systems for FPs are being developed. This review focuses on recent innovative nanodelivery approaches for FP drugs that display therapeutic promise. We summarize established, clinically useful FP prodrug strategies, including capecitabine, which exploit tumor-specific enzyme expression for optimal anticancer activity. We then describe the use of FP DNA-based polymers (e.g., CF10) for the delivery of activated FP nucleotides as a nanodelivery approach with proven activity in pre-clinical models and with clinical potential. Multiple nanodelivery systems for FP delivery show promise in CRC pre-clinical models and we review advances in albumin-mediated FP delivery, the development of mesoporous silica nanoparticles, emulsion-based nanoparticles, metal nanoparticles, hydrogel-based delivery, and liposomes and lipid nanoparticles that display particular promise for therapeutic development. Nanodelivery of FPs is anticipated to impact CRC treatment in the coming years and to improve survival for cancer patients.

## 1. Introduction

Cancer is the second leading cause of death worldwide, with 10 million deaths attributed to cancer in 2020 [1]. Cancers of the gastrointestinal (GI) tract account for more than one-third of cancer-related deaths [2], with colorectal cancer (CRC) alone responsible for >930,000 deaths in the current year. CRC incidence is rapidly increasing, and by 2040 it is anticipated to increase to >3.2 million new cases causing >1.6 million deaths [3].

Colorectal cancer (CRC) is a disease caused by a combination of genetic, environmental, and lifestyle factors. The common risk factors include inherited genetic mutations, obesity, heavy alcohol consumption, smoking, inflammatory bowel disease, and certain dietary habits. Although the overall rates of CRC in people over 50 years old have decreased, there has been a continuous increase in the prevalence of early-onset CRC (EOCRC), CRC diagnosed in individuals younger than 50 years old [4]. Though EOCRC is generally diagnosed at more advanced stages with higher metastatic potential, individuals’ overall survival probability is higher than older adults due to an increased tolerance to chemotherapy and better performance status [5,6]. In a recent study, incidence rates of EOCRC were found to be surging, whereas average-onset colorectal cancer (AOCRC) rates are decreasing [7].

Racial disparities are also observed for CRC in the US and around the world. In the US, CRC rates are highest in non-Hispanic Blacks in comparison to other racial/ethnic groups, such as non-Hispanic Whites or Asians, and the prevalence of risk factors, including a lack of physical activity, high consumption of red and processed meats, and smoking, play a vital role [8,9].

The mortality of CRC results almost exclusively from metastatic disease with the liver being the most frequent site of metastasis and the thorax and peritoneum also frequent sites of metastatic progression in colon cancer [10]. Synchronous liver metastases are those detected at diagnosis or within 6 months post-operatively and occur in approximately one-fourth of CRC patients, while up to 70% of CRC patients will eventually develop a metachronous metastatic disease [11]. Metastatic CRC (mCRC) is highly lethal, with 3% 60-day mortality [12] and <14% 5-year survival rates [13].

### 1.1. FP-Based Combination Chemotherapy for CRC

**Figure 1:**
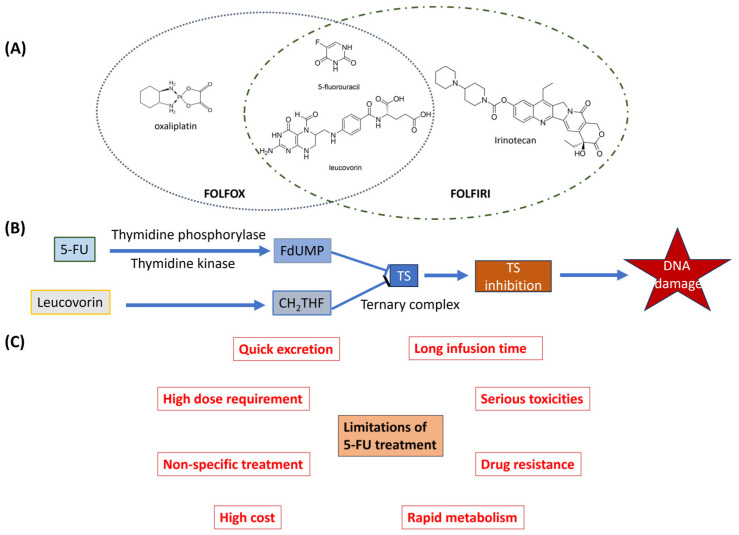
(**A**) Structure of 5-FU and leucovorin and components of FOLFOX and FOLFIRI. (**B**) Schematic demonstration of TS inhibition of 5FU resulting in DNA damage. (**C**) Limitations of 5FU treatment.

While the mortality statistics for mCRC are sobering and the percentage of patients diagnosed with localized disease that later relapse with mCRC remains high, the present mortality and relapse rates also represent a considerable advance relative to the introduction of 5-Fluorouracil (5-FU)-based chemotherapy ~60 years ago. 5-FU administered together with the reduced folate leucovorin (5-FU/LV) was proven to improve disease-free survival and overall survival in adjuvant chemotherapy administered post-surgically in CRC patients with locally advanced (stage III), non-metastatic CRC [14]. The current standard of care for locally advanced non-metastatic CRC includes adjuvant chemotherapy with 5FU/LV combined with oxaliplatin in the FOLFOX regimen. For mCRC, either FOLFOX or the related regimen in which 5-FU/LV is combined with irinotecan (FOLFIRI) are front-line chemotherapy regimens used to treat non-resectable liver metastatic disease or used for neo-adjuvant chemotherapy to enable resection (Figure 1).

FOLFOX is more effective in treating advanced colon cancer patients, with a response rate of 53% and progression-free survival of ~9 months. In comparison, the response rate for 5-FU/LV alone is only 22%, with a progression-free survival of ~6 months [15]. FOLFIRI provides a progression-free survival of 7 months and an overall response rate of 39%, but it does produce more adverse side effects, such as nausea, diarrhea, and neutropenia. On the other hand, FOLFOX patients are more likely to experience neuropathy [16].

While the use of 5-FU-based chemotherapy regimens has contributed significantly to improved survival in mCRC and reduced the likelihood of recurrent disease for locally advanced CRC, current regimens have serious limitations that necessitate the development of next-generation fluoropyrimidine (FP) drugs and nanodelivery systems [17,18]. A major driving force for developing new FPs and improved nanodelivery systems is that the improved outcomes achieved with current regimens have resulted from the optimization of scheduling and delivery for 5-FU and the identification of drug combinations that include 5-FU that display improved survival with an acceptable toxicity profile. For example, 5-FU is frequently delivered using extended infusion over approximately 46 h to reduce serious systemic toxicities associated with its bolus administration and counter its short half-life (around 10–20 min). The upward trajectory in survival achieved through these optimization approaches is, however, unlikely to be sustainable. Nanodelivery approaches may also counter the low bioavailability, rapid metabolism, and moderate selectivity for cancerous cells that are some of the disadvantages associated with 5-FU treatment [19].

### 1.2. Mechanistic Targets of FP Chemotherapy

The main target of 5-FU’s anticancer activity is thymidylate synthase (TS), which plays a crucial role in DNA replication. The inhibition of TS occurs through the formation of a ternary complex between folinic acid (Leucovorin; LV), a reduced folate co-factor that binds to TS, and Fluorodeoxyuridylte (Fluoro-2′-deoxyuridine-5′-O-monophosphate (FdUMP)), a 5-FU metabolite that irreversibly inhibits TS enzymatic activity. The inhibition of TS leads to an increased misincorporation of 2′-deoxyuridine-5′-triphosphate (dUTP) in DNA, causing Topoisomerase 1 (Top1)-mediated DNA damage [20].

The correlation between TS (thymidylate synthase) levels and the response to 5-FU (fluorouracil), other FPs (fluoropyrimidines), or TS inhibitors is intricate. Elevated levels of TS contribute to resistance, as it requires more Fluorodeoxyuridylate (FdUMP) for TS inhibition. On the other hand, very low levels of TS decelerate cell proliferation, which is crucial for replication-dependent DNA damage [21]. A major limitation of 5-FU treatment is that only a small percentage of the administered drug (less than 5%) is converted to FdUMP and DNA-directed metabolites in the human body. Most 5-FU is degraded in the liver or excreted in the urine without undergoing any metabolic changes [22].

Among all therapeutic approaches, oral drug delivery is the simplest and most promising technique for treating CRC. The effectiveness of oral drug delivery depends on its absorption and metabolism in the liver, as well as its stability in the system [23]. Factors that can significantly affect this include varying pH in the GI tract, the presence of degradable enzymes in the small and large intestines, and variations between patients [24]. To overcome these challenges, drugs must be protected in the form of prodrugs, micelles, liposomes, and nanoparticles before delivery to the specific region [25]. This review discusses several clinically approved prodrugs for delivering 5-FU to treat CRC.

## 2. Prodrugs of 5-FU for Oral Bioavailability and Tumor-Specific Activation

### 2.1. Oral 5-FU Prodrugs

Prodrugs, which harbor masked pharmacological activity that is recovered upon bioconversion in the body are associated with more suitable pharmacokinetic properties (such as increased bioavailability, improved permeability, and reduced toxicity) and beneficial administration routes (including oral, intravenous, and inhalation administration) than parent drugs, making this strategy an ideal option against the epidemic [26]. The antitumor activity of 5-FU requires intravenous administration, which often requires long infusions leading to lengthy hospitalizations, and these add significantly to the cost of care. In principle, the oral delivery of 5-FU would eliminate the need for long infusion, however, the direct oral administration of 5-FU is not feasible due to its phosphorylation via OPRT, which promotes ribonucleotide-mediated GI toxicity. To overcome this limitation, 5-FU prodrugs that were orally available were developed [27], of which capecitabine is the most widely used clinically (Table 1).

Capecitabine. The design concept for capecitabine was the development of an orally available precursor or prodrug of 5-FU that would require activation by enzymes expressed at relatively higher levels in malignant tissue compared to sensitive, non-malignant tissues [28]. Capecitabine (N4-pentyloxycarbonyl-5′-deoxy-5-fluorocytidine) is a 5-fluorocytidine analog in which the N4 amino group is protected via a pentyl carbamate linkage. After oral administration, capecitabine enters the blood and is metabolized in the liver where high carboxylesterase levels cleave the carbamate linkage. Further enzymatic activation by cytidine deaminase and thymidine phosphorylase (TP) release 5-FU. The relatively higher TP levels expressed in many tumors in principle result in the selective activation of capecitabine in tumor tissue [29]. However, toxicity and efficacy data for capecitabine and 5-FU and associated combination regimens (e.g., FOLFOX, CAPOX) are similar.

Tegafur, Ftorafur, S1. Other oral prodrugs of 5-FU that are in clinical use are based on Tegafur, and these include S1, which is used in Japan and other nations for GI malignancies and other cancer types, and Ftorafur. Tegafur is an orally available 5-FU prodrug with a furanyl moiety that is activated by CYP2A6 in the liver to release 5-FU [30,31]. In S1, Tegafur is combined with potassium oxonate (O(XO)) to inhibit pyrimidine phosphoribosyltransferase (OPRTase) and 5-chloro-2,4-dihydroxypyridine (CDHP), which inhibits dihydropyrimidine dehydrogenase (DPD) that is responsible for 5-FU breakdown. S1 is used for the treatment of stomach and esophageal cancers in Asia [32]. Ftorafur is a combination of Tegafur and uracil, which competes with 5-FU for DPD-mediated degradation resulting in increased 5-FU [31].

Doxifluridine. Doxifluridine includes a 5′-deoxyribose sugar and releases 5-FU through thymidine phosphorylase (TP) enzymatic activity, which is expressed at elevated levels in in many tumors [33] Although doxifluridine showed acceptable activity for colorectal cancers in its first clinical trial via i.v. administration, its toxic effects on the nervous and cardiovascular systems prevented this mode of administration [34].

### 2.2. Mutual Prodrugs of 5-FU

**Figure 2:**
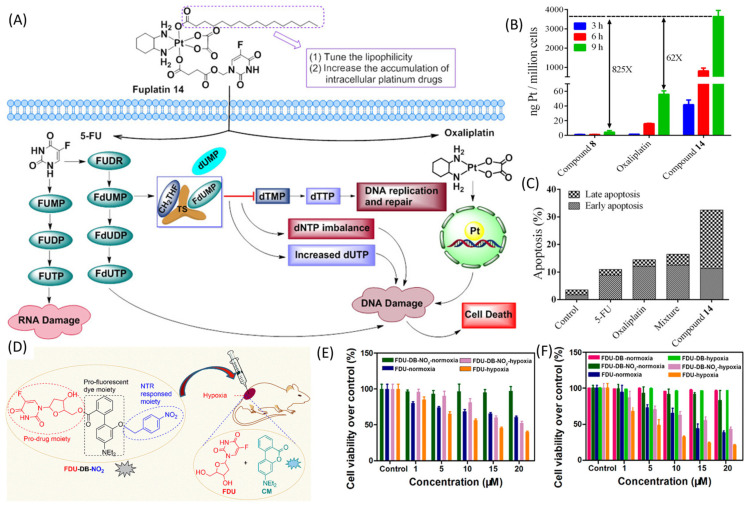
(**A**) Schematic depiction of Fuplatin design and mechanism of activity. (**B**) Determination of Pt accumulation in HCT116 cells after treatment with different concentrations of Pt-containing drugs. (**C**) Percentage apoptosis measurement after treatment with equimolar amounts of 5-FU, oxaliplatin, and their mixture. (**D**) Structure and release mechanism of prodrug FDU-DB-NO2 under hypoxia condition. Cell viability of MCF-7 cells after treatment with FDU-DB-NO2 and other controls under normoxic and hypoxic conditions for (**E**) 24 h and (**F**) 48 h [40,42]. [Reproduced with permission].

Mutual prodrugs are designed to release two drugs through the enzymatic hydrolysis of a linker [35], with an example being sulfasalazine in which sulfapyridine is linked to 5-amino salicylic acid through an azo linker that may be cleaved by the gut microbiome resulting in an FDA-approved treatment for ulcerative colitis [36]. A similar strategy was used to create combined 5-FU prodrugs [35], including 5-FU/HDAC inhibitors [37], 5-FU/deoxypodophyllotoxin [38], and other 5-FU combinations, including 5-FU/oxaliplatin [39], which delivers two of the three drug components included in the widely used FOLFOX regimen. To achieve tumor-targeting specificity, 5-FU was linked to an RGD peptide, through cleavable disulfide linkage, targeting integrin α_v_β_3_ [40]. Among these α_v_β_3_ integrin-overexpressing cancer cells A549, MDA-MB-321, and PC3, the 5-FU conjugate showed profound activity in A549 cells only (Figure 2).

### 2.3. Radiation- and Hypoxia-Mediated 5-FU Release

5-FU is used together with radiation for the treatment of some malignancies, including head-and-neck cancer and rectal cancer. In principle, improved specificity for tumors could be achieved using 5-FU prodrugs, where the release of 5-FU was stimulated by ionizing radiation specifically targeting tumor tissue. Several groups have synthesized such radiation-activated 5-FU prodrugs, including N1-substituted 5-FU prodrugs and N1-C5-linked 5-FU dimers that release 5-FU upon interaction with a radiation-induced free radical release mechanism [41]. A characteristic of many tumors, particularly large primary tumors, is hypoxia, and tumor specificity for drug activation can be achieved by conjugating a drug to permit hypoxia-induced drug release. Under hypoxic conditions, an elevated expression of reductase enzymes, including nitroreductase, may occur and FdU conjugated to the nitrobenzyl group may be selectively released in hypoxic tumors [42,43]. A theranostic prodrug, FDU-DB-NO2, showed the concurrent release of FdU and the fluorescent analog coumarin under hypoxia, leading to the detection as well as inhibition of the tumor [42]. The selective activation of the prodrug under hypoxia and minimum toxicity at a normoxic condition showed great potential for cancer treatment (Figure 2).

## 3. FdUMP Prodrugs Enhance TS Inhibition

The metabolism of 5-FU is similar to uracil, and 5-FU-derived metabolites compete with uracil-derived metabolites as substrates for multiple enzymes with minimal differences in catalytic efficiency with one notable exception: thymidylate synthase (TS) [44,45]. TS converts uridylate (dUMP) to thymidylate (TMP) via a reductive methylation reaction in which 5,10-methylene tetrahydrofolate serves as a co-factor.

### 3.1. ProTides and Development of NUC-3373

The importance of TS inhibition by FdUMP for the antitumor activity of 5-FU resulted in the development of FdUMP prodrugs for which NUC-3373 is presently in clinical development. NUC-3373 is a phosphoramidite prodrug of FdUMP that enters cells independent of nucleoside transporters and then undergoes metabolism to release FdUMP, bypassing a requirement for enzymatic phosphorylation by thymidine kinase. Strong TS inhibition is detected in cancer cells treated with NUC-3373 and the compound is in phase 1 clinical trials [46]. NUC-3373 is one example of a “ProTide” strategy in which biologically active nucleosides are delivered with the hydroxyl of the 5′-monophosphate blocked by an aromatic group or an amino acid ester [47]. Other strategies to deliver FdUMP include the preparation of sulfonyl-containing FdU phosphotriester and phosphoramidite derivatives that undergo β-elimination to release FdUMP [48].

### 3.2. Dinucleosides and Mutual Prodrugs

Nucleosides and nucleotides play vital roles in tumor treatment, and their prodrugs are developed successfully to elevate their oral absorption leading to greater activity. Cyclic dinucleotides (CDNs) acted as potential cancer vaccine adjuvants inducing antitumor activity through immune cell activation. However, due to their rapid clearance and limited cellular permeability, prodrugs were developed with improved activity [49]. A dithioethanol (DTE)-based dCDN prodrug (DTE-dCDN) displayed prompt decomposition to release the parent dCDN in the presence of GSH or DTT [50]. Free dCDN stimulated the GAS-STING pathway with the production of proinflammatory cytokines. Another dinucleotide S110 consists of 5-Azacytidine, a DNA methylation inhibitor, and a deoxyguanosine was found to be effective in DNA methylation and inducing p16 expression [51]. S110 was delivered through i.p and s.c injections and dissociated into individual nucleotides resulting in the retardation of tumor growth in mice.

### 3.3. F10 and CF10: DNA-Based Fluoropyrimidine Polymers

**Figure 3:**
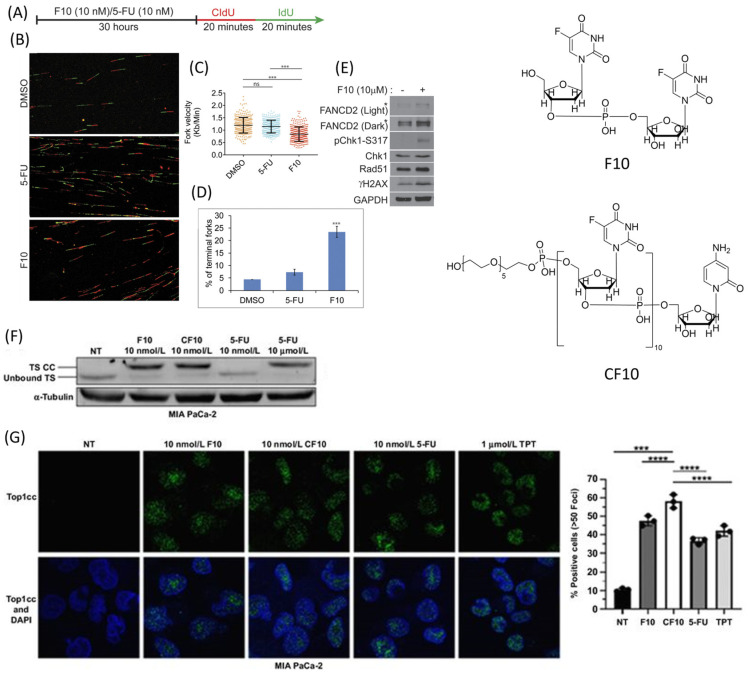
DNA-based fluoropyrimidine polymers dual target TS and DNA topoisomerase 1 (Top1). (**A**) Treatment of HCT-116 cells with equal concentrations of F10 and 5-FU followed by CldU and IdU incorporation. (**B**) Analysis of corresponding DNA fiber images. Significant reductions in (**C**) fork velocity and (**D**) terminal forks by F10 (10 nM) than 5-FU (10 nM). (**E**) Evaluation of replication stress-associated proteins of HCT-116 cells after 2 h of exposure to F10 (10 μm). (**F**) Western blot showing the detection of TS classic complex (TS CC) formation after treatment with F10, CF10, and 5-FU at specified concentrations for 24 h. (**G**) Representative immunofluorescence images with an antibody specific for Top1 cleavage complexes (Top1cc—green with DAPI (blue) nuclear staining) at indicated concentrations of F10, CF10, and 5-FU for 24 h and Topotecan for 1 h, with quantification graphs to the right [54,56]. (***, *p* < 0.001, ****, *p* < 0.0001) [Reproduced with permission].

Since the DNA-directed FP metabolites FdUMP and FdUTP are primarily responsible for the antitumor activity of 5-FU and FP drugs, DNA-based FP polymers such as F10 and CF10 that directly release FdUMP have the potential to display improved potency relative to monomeric FPs (Figure 3). The prototype FP polymer F10 is a single-stranded DNA consisting of 10 FdU nucleosides, and studies in cancer cell lines deficient in OPRTase and TK cytotoxicity [52] showed a reduced dependence on enzymes required for the metabolic activation of 5-FU and FdU consistent with the cell uptake of the multimer and intracellular release of FdUMP [52,53]. Overall, the potency advantage of F10 across the NCI 60 cell-line screen was 338-fold greater than 5-FU [54]. With folic acid, conjugation-improved anticancer activity and specificity for colon cancer cells were achieved [55]. A second-generation FP polymer, CF10, includes AraC at the 3′-terminus and displays increased stability to exonucleolytic degradation relative to F10, and is more potent to CRC cell lines resulting from the dual-targeting of thymidylate synthase (TS) and DNA topoisomerase 1 (Top1) [56]. Recent studies demonstrated improved antitumor activity for CF10 relative to 5-FU in a mouse model of primary colon cancer and in a rat model of colorectal cancer liver metastases. Nano-properties improved the antitumor activity of CF10 associated with the extended detection of FdU and FUMP in plasma consistent with improved PK properties relative to 5-FU and other monomeric FPs.

### 3.4. FdU DNA Polymer Conjugates

FdU5 DNA polymers were synthesized as conjugates with cholesterol, palmitic acid, GalNAc, PEG, and folic acid, with only the folic acid conjugation displaying improved potency in cancer cell lines [57]. DNA hairpins containing FdU nucleotides also have therapeutic potential for FP delivery, and the increased structural complexity of the DNA hairpin enabled the inclusion of other drugs, such as doxorubicin, or biological modulators, such as netropsin [58,59] and curcumin [60].

### 3.5. FdU DNA Aptamers

Aptamers specifically bind to certain cell surface proteins to deliver drug molecules. During degradation to nucleosides, FdU is released from a modified aptamer containing 30 units of FdU in a controlled manner, which results in decreased cell proliferation and increased cell death. The synthesis of a modified aptamer was achieved enzymatically in a single step [61].

### 3.6. DNA Origami

A significant advancement in DNA nanostructures is the development of DNA origami by P. Rothemund, where a long, single-stranded scaffold is folded using numerous short ‘staples’ strands to generate 2D and 3D shapes [62]. Td, a tetrahedral DNA nanostructure composed of four short complementary DNA strands, is another promising drug delivery vehicle that can hybridize in a single-step process. Like DNA origami, Td has successfully delivered anticancer drugs like DOX and therapeutic nucleic acids [63,64]. Therapeutic oligonucleotides composed of FdU nucleotides were added to Td and origami staples followed by their modification with cholesterol to improve nanocarrier uptake. The modifications maximized the number of synthesized strands without affecting the structures and evolved as an effective system for the delivery of FdU_n_ oligonucleotides, inducing higher in vitro cytotoxic and anti-proliferative effects on colorectal cancer cells than the conventional drugs 5-FU and FdU [65]. However, the presence of cholesterol in DNA nanoscaffolds boosted their antiproliferative action.

## 4. Nanodelivery Systems

**Figure 4:**
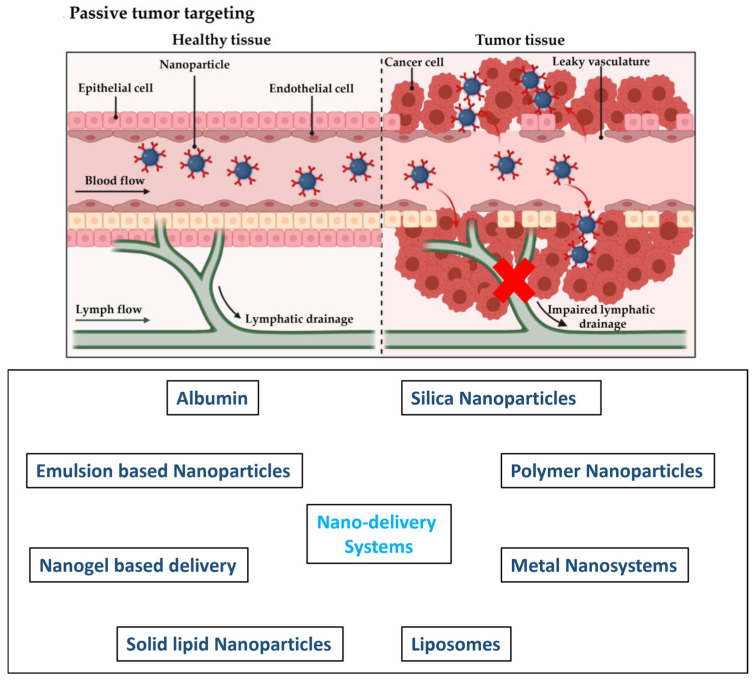
Pictorial demonstration of passive tumor targeting of nanoparticles through the EPR effect [67] and examples of 5-FU nanodelivery systems discussed herein. [Reproduced with permission].

Nanotechnology is a valuable tool for improving cancer treatment [66]. Nanocarriers less than 500 nm in size can improve the biological activity of their payloads. They rely on the enhanced permeability and retention (EPR) effect for cancer therapy, which enables passive targeting and the long-term retention of nanoparticles at the tumor site [67] (Figure 4). Nanomaterials have become increasingly important in medicine and cancer research [68]. As a promising drug delivery system, different types of nanoparticles were explored to deliver 5-FU and its prodrugs for the treatment of colorectal cancer. Here, we discuss various nanodrug delivery systems, such as silica nanoparticles, albumin, polymeric nanoparticles, metal-based nanoparticles, liposomes, and lipid nanoparticles, extensively used to transport 5-FU and other fluoropyrimidine-based drugs for the treatment of colorectal cancer (Figure 4).

### 4.1. Albumin

Albumin-based nanoparticles are an effective drug delivery system as they are biodegradable and biocompatible, and tend to accumulate in cancer cells due to the presence of gp60 and SPARC receptors [69]. 5-FU albumin nanoparticles (5FU-A1) were 16-times more effective than free 5-FU in treating MCF-7 cancer cells [70] and were found to induce cell death through apoptosis. Importantly, 5FU-A1 did not show any significant cytotoxicity to normal HUVEC cells. 5FU-A1 acted as a P-gp inhibitor resulting in reduced resistance and increased sensitivity in the treatment of cancer cells. Albumin is also used as a transporter to deliver poorly water-soluble drugs into cells. Lipid-conjugated floxuridine homomeric oligonucleotides were initially self-assembled into a micelle, and then formed a complex with endogenous serum albumin in situ after injection into a cell. The resulting LFU20/albumin complex led to a decrease in cell proliferation [71]. Though albumin-based NPs served as a promising career for targeted delivery of 5-FU with minimum side effects, its long-term effectiveness need to be evaluated as a drug delivery system.

### 4.2. Mesoporous Silica Nanoparticles

**Figure 5:**
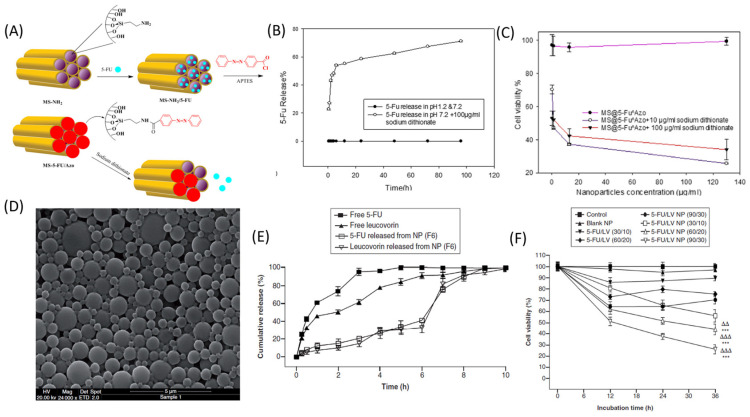
(**A**) Schematic demonstrating the synthesis of MS@5-FU^Azo and the release of 5-FU in the presence of a reducing agent (sodium dithionate). (**B**) %Release of 5-FU from MS@5-Fu^Azo at pH 1.2, 7.2, and in the presence of 100 μg/mL sodium dithionate, pH 7.2. (**C**) Cell viability analysis after incubation with MS@5-Fu^Azo for 48 h on HT-29 cells. (**D**) SEM image of 5FU-LV NPs. (**E**) Cumulative release of 5FU and LV from NPs in the presence of gastrointestinal fluids. (**F**) Cell viability of different doses of 5FU, LV, and 5FU-LV NPs after incubation with HT-29 cells. *** *p* < 0.001 in comparison to control and blank NPs. ΔΔ *p* < 0.01, ΔΔΔ *p* < 0.001 in comparison to same dose of free 5-FU and LV. [74,78]. [Reproduced with permission].

Mesoporous silica nanoparticles (MSNs) can release drugs via tunable diffusion to produce a more favorable PK profile. By surface derivatization with amine groups, a high loading capacity for 5-FU of 28.89% was achieved [72], while other methods yielded loading in excess of 95%. MSN loading with 5-FU resulted in controlled release profiles over 72 h and displayed selective cytotoxicity to cancer cell lines relative to non-malignant cell lines [73]. To enable the specific release of 5-FU in the colon, amino-functionalized MSN was modulated using an azobenzene derivative as a gatekeeper that prevented 5-FU release, unless activated by azoreductase from normal colon flora-stimulated release (Figure 5) [74]. The specificity of MSN-mediated delivery of 5-FU for EGFR^+^ colon cancer was achieved using EGF-grafted MSNs [75], while RGD conjugation of 5-FU-loaded MSNs improved anticancer efficacy [76]. Affixing carbon dots derivatized with folic acid to MSNs was also shown to improve cancer-specific targeting for the delivery of 5-FU [77]. Among these MSN-based delivery methods, RGD-conjugated MSNs emerged as potential delivery systems with the specific delivery of 5-FU.

### 4.3. Polymer Nanoparticles

**Figure 6:**
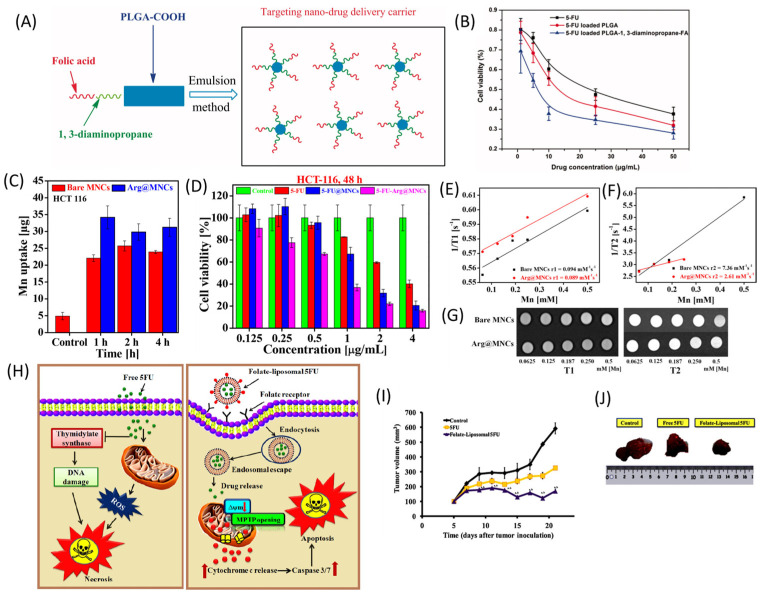
(**A**) Schematic demonstration of PLGA-1, 3-diaminopropane-folic acid nanodrug delivery system [81]. (**B**) Comparison of the cytotoxicity effect of 5-FU, 5-FU-PLGA, and 5-FU-PLGA-1, 3-diaminopropane-folic acid nanoparticles to HT-29 cells [81]. (**C**) Cellular uptake of bare MNCs and Arg@MNCs in HCT 116 cells [83]. (**D**) Cytotoxicity effect of 5-FU, 5-FU@MNCs, and 5-FU-Arg@MNCs on HCT 116 [83]. (**E**) T1 and (**F**) T2 vs. Mn concentration for bare MNCs and Arg@MNCs [83]. (**G**) T1-weighted MR images (left) and T2-weighted MR images (right) of the MNC and Arg@MNC aqueous solutions at various Mn concentrations [83]. (**H**) Schematic representation of action mechanism of 5FU and folate-liposomal 5FU in HeLa cell. [84]. (**I**) Changes in tumor volume and (**J**) photographs of tumor after treatment with control, 5-FU, and folate liposomal 5-FU [84]. [Reproduced with permission].

Biodegradable and biocompatible polymeric nanoparticles emerged as an effective vehicle for cancer drug delivery providing sustained and prolonged drug release. PLA-PEG NPs were optimized for 5-FU delivery and achieved a 51% encapsulation efficiency, were orally bioavailable, and displayed improved pharmacokinetic properties relative to free 5-FU [79]. To provide targeting for colon cancer, PLGA NPs were prepared with 5-FU encapsulation and the NPs were derivatized with EGF [80]. 5-FU-encapsulated PLGA-1,3-diaminopropane-folic acid nanoparticles demonstrated a dramatic decrease in cell viability due to a folic acid-mediated targeted effect on HT-29 cancer cells (Figure 6) [81].

### 4.4. Emulsion-Based Nanoparticles

A modified double-emulsion (W1/O/W2) solvent-evaporation method generated 5-FU/LV-loaded Eudragit S100 nanoparticles that showed a synergistic drug combination effect against colorectal cancer. At colon pH, rapid drug release due to the complete dissolution of the NP matrix displayed significant cytotoxicity to CT-26 and HT-29 cell lines in comparison to the free drug combination [78]. An oral nanodelivery system containing OXA and 5-FU was developed by using a deoxycholic acid derivative (N-α-deoxycholyl-L-lysyl-methylester, DCK) (OXA/DCK) that formed an ion-pairing complex with OXA and acted as a permeation enhancer. The water-in-oil-in-water nanoemulsion was used for oral administration to the colorectal adenocarcinoma cell (CT26)-bearing mouse model and showed a 9.19- and 1.39-fold increase in bioavailability compared with that of free OXA and 5-FU, respectively, leading to the enhanced inhibition of tumor growth with an improved pharmacokinetic effect [82]. In mice with colorectal cancer, the addition of the drug resulted in a 73.9% reduction in tumor volume, compared to the control group and the oral OXA and 5-FU groups.

### 4.5. Metal Nanoparticles

Manganese oxide nanocuboids (MNCs) were functionalized with arginine, loaded with 5-FU, and used for drug delivery and in theranostic applications with Mn^2+^ paramagnetic effects on ^1^H_2_O relaxivity in magnetic resonance detection (Figure 6) [83]. DNA hairpin gold nanorods functionalized with a DNA hairpin containing FdU nucleotides showed promising activity with the release of the DNA hairpin stimulated by laser irradiation at 808 nm [85,86].

The conjugation of 5-FU to the surface of selenium nanoparticles (5FU-SeNPs) not only exhibited an enhanced cellular uptake of SeNPs through endocytosis leading to the effective inhibition of cancer cell growth, but also showed great selectivity between normal and cancer cells [87]. 5FU-SeNPs-induced apoptosis was dependent on reactive oxygen species (ROS) generation and caspase pathways. However, selenium toxicity could be a limitation for the administration of these NPs, which needs further evaluation.

Host–guest chemistry was used to generate a nanocarrier for the delivery of 5-FU and microRNA-34a (miR-34a(m)), a key regulator of tumor advancement that can also hinder tumor progression and metastasis. As a host molecule, multiple ß-cyclodextrin (CD)-attached QD nanoparticles were used with a adamantane (ADA)-based TCP1 peptide-targeting ligand (TCP1) as a guest molecule [88]. A sustained release of 60% over the first 12 h from TCP1-CD-QDs/5-FU-miR-34a(m) resulted in a decreased cell viability of 49.9% ± 3.2%, higher than that of only 5-FU treatment (77.1% ± 5.5%). A synergistic effect of 5-FU and miRNA-34a was observed causing tumor cell death and reduced drug resistance with minimum tumor cell migration.

### 4.6. Hydrogel-Based Delivery

The extended release of 5-FU using a biodegradable nanogel was achieved by gamma irradiation of acrylic acid monomers and gelatin, and the resulting nanogel showed anticancer activity upon intra-peritoneal injection [89]. A micelle/hydrogel composite was developed by mixing 5-FU-loaded gelatin with hydrazide functionality (Gel-ADH) and a curcumin-loaded Pluronic F127-benzaldehyde (PF127-CHO) [90]. The composite showed a pH-dependent release pattern for 5-FU and curcumin. The synergistic effect of 5-FU and curcumin led to profound cytotoxicity in HT-29 cells reflecting curcumin-induced necrosis and 5-FU-induced apoptosis. Degradable hydrogels with a gamma radiation-mediated release of 5-FU showed promising results, but enzymatic responsive degradation with a concurrent release of 5-FU could be an ideal hydrogel-based delivery system.

### 4.7. Liposomes and Lipid Nanoparticles

Lipid-based nanocarriers are the least toxic for in vivo use among all nanodelivery systems and show significant progress in DNA/RNA delivery [91]. 5-FU-loaded liposomes were prepared by dissolving a lipid mixture (DPPD/cholesterol/FA-PEG-DSPE) in CHCl_3_ and using a thin-film hydration technique (Figure 6). Folic acid conjugation of PEG-DSPE was used to increase colon cancer specificity [84]. Solid lipid nanoparticles loaded with 5-FU (5FU-SLN4) showed a greater inhibition of HCT116 cell growth and a 2.4-fold reduction in the IC_50_ value than free 5-FU treatment [92]. 5FU-SLN4 also impeded HER2 expression, responsible for CRC progression [93] in mouse-bearing HCT116 tumor cells. The potential of 5-FU or fluoropyrimidine-based lipid nanoparticles with different targeting agents need to be evaluated as future drug delivery systems for colorectal cancer.

While the nanoparticle-based delivery of 5FU and other fluoropyrimidine drugs showed immense potential, the toxic effects and challenges of using nanoparticles, especially metal nanoparticles, could not be ignored. Being small in size, they penetrate physiological barriers, causing several side reactions, such as oxidative stress, cell damage, and organ injury [94]. The toxicity associated with nanoparticles varies with their composition, size, surface functionalities, and their treatment time [95]. Mostly, their pharmacokinetics and pharmacodynamics studies are overlooked, and the long-term effects of nanoparticle treatments are yet to be explored [96,97]. Despite having side effects in the application of some nanoparticles, there are examples of nanoparticles, such as Doxil, ONPATTRO, Hensify, and Abraxan, used for clinical treatments [98].

## 5. Conclusions and Future Perspectives

In this review, we discussed the survival rate and causes of CRC and the current treatment methods emphasizing cytotoxic chemotherapy, which is widely used both in the adjuvant setting and for the treatment of metastatic disease [99]. The anticancer drug 5-FU has been widely used to treat CRC, and it is central to the current combination chemotherapy regimens in clinical use [100]. As a consequence of its importance for CRC treatment, several prodrugs of 5-FU and other fluoropyrimidine-based nanomedicines are being developed. 

We highlighted the current prodrugs of 5-FU, along with their design strategies for stimulated release, method of action, and extent of clinical use. As Fluorodeoxyuridylate is central to TS inhibition, prodrugs are being developed with compelling delivery strategies and release studies. Tumor microenvironment biomarkers, such as hypoxia, acidic pH, an oxidative and/or reductive environments, and different enzymes are used as triggers for drug release to the tumors. Fluoropyrimidine-based drugs F10 and CF10 have shown outstanding effects in the treatment of colorectal cancer and could be a future CRC treatment strategy for clinical use. Another promising method of delivery discussed here is the use of nanoparticles. After analyzing various studies, it has been found that using nanocarriers to guide the delivery of chemotherapeutic agents has significantly improved their effectiveness. Polymer nanoparticles and lipid nanoparticles have improved the solubility of certain agents in water, which in turn makes them more accessible to the body. These particles also help to prolong the time that the agents remain in the bloodstream, target specific areas of the body, and are more easily absorbed by cells. As a result, they have proven to be more effective in fighting cancer.

Although progress has been made, the clinical translation of these agents is still challenging. Prodrugs with improved pH sensitivity are required for specific delivery to tumor cells in response to the tumor microenvironment. Along with the evolution of prodrugs, their delivery strategies must be optimized to render targeted delivery. It is also crucial to refine the chemical structures of the prodrugs and nanocarriers to minimize off-target toxicity, optimize pharmacokinetics and pharmacodynamics, and improve tumor specificity. 

## Figures and Tables

**Table 1:** Prodrugs of 5-FU.

Name	Structure	Importance
Capecitabine	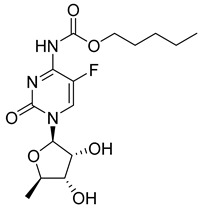	activation by enzyme carboxylesterase
Tegafur	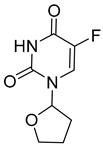	activated by CYP2A6 in the liver to release 5-FU
S-1	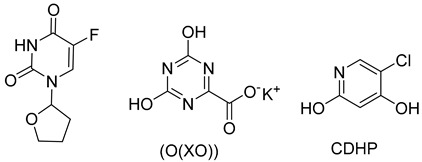	inhibits dihydropyrimidine dehydrogenase (DPD), which is responsible for 5-FU breakdown
Ftorafur	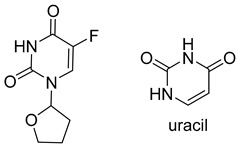	competes with 5-FU for DPD-mediated degradation resulting in increased 5-FU
Doxifluridine	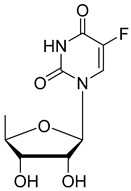	releases 5-FU through thymidine phosphorylase (TP) enzymatic activity
NUC-3373	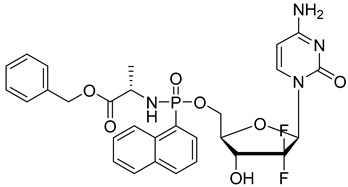	phosphoramidite prodrug of FdUMP enters cells independent of nucleoside transporters

## Data Availability

Not applicable.

## References

[1] American Cancer Society (2024). The Global Cancer Burden. https://pressroom.cancer.org/GlobalCancerStatistics2024.

[2] Arnold M., Abnet C.C., Neale R.E., Vignat J., Giovannucci E.L., McGlynn K.A., Bray F. (2020). Global Burden of 5 Major Types of Gastrointestinal Cancer. Gastroenterology.

[3] Xi Y., Xu P. (2021). Global colorectal cancer burden in 2020 and projections to 2040. Transl. Oncol..

[4] Adigun A.O., Adebile T.M., Okoye C., Ogundipe T.I., Ajekigbe O.R., Mbaezue R.N., Okobi O.E. (2023). Causes and Prevention of Early-Onset Colorectal Cancer. Cureus.

[5] Medici B., Riccò B., Caffari E., Zaniboni S., Salati M., Spallanzani A., Garajovà I., Benatti S., Chiavelli C., Dominici M. (2023). Early onset metastatic colorectal cancer: Current insights and clinical management of a rising condition. Cancers.

[6] Willauer A.N., Liu Y., Pereira A.A., Lam M., Morris J.S., Raghav K.P., Morris V.K., Menter D., Broaddus R., Meric-Bernstam F. (2019). Clinical and molecular characterization of early-onset colorectal cancer. Cancer.

[7] Chen Y., He L., Lu X., Tang Y., Luo G., Chen Y., Wu C., Liang Q., Xu X. (2023). Causes of death among early-onset colorectal cancer population in the United States: A large population-based study. Front. Oncol..

[8] Murphy C.C., Wallace K., Sandler R.S., Baron J.A. (2019). Racial disparities in incidence of young-onset colorectal cancer and patient survival. Gastroenterology.

[9] Zahnd W.E., Gomez S.L., Steck S.E., Brown M.J., Ganai S., Zhang J., Arp Adams S., Berger F.G., Eberth J.M. (2021). Rural-urban and racial/ethnic trends and disparities in early-onset and average-onset colorectal cancer. Cancer.

[10] Riihimaki M., Hemminki A., Sundquist J., Hemminki K. (2016). Patterns of metastasis in colon and rectal cancer. Sci. Rep..

[11] Wang J., Li S., Liu Y., Zhang C., Li H., Lai B. (2020). Metastatic patterns and survival outcomes in patients with stage IV colon cancer: A population-based analysis. Cancer Med..

[12] van der Kruijssen D.E.W., Elias S.G., Vink G.R., van Rooijen K.L., t Lam-Boer J., Mol L., Punt C.J.A., de Wilt J.H.W., Koopman M., CAIRO4 Working Group (2021). Sixty-Day Mortality of Patients With Metastatic Colorectal Cancer Randomized to Systemic Treatment vs Primary Tumor Resection Followed by Systemic Treatment: The CAIRO4 Phase 3 Randomized Clinical Trial. JAMA Surg..

[13] Shin A.E., Giancotti F.G., Rustgi A.K. (2023). Metastatic colorectal cancer: Mechanisms and emerging therapeutics. Trends Pharmacol. Sci..

[14] Arkenau H.T., Bermann A., Rettig K., Strohmeyer G., Porschen R., Arbeitsgemeinschaft Gastrointestinale Onkologie (2003). 5-Fluorouracil plus leucovorin is an effective adjuvant chemotherapy in curatively resected stage III colon cancer: Long-term follow-up results of the adjCCA-01 trial. Ann. Oncol..

[15] Neugut A.I., Lin A., Raab G.T., Hillyer G.C., Keller D., O’Neil D.S., Accordino M.K., Kiran R.P., Wright J., Hershman D.L. (2019). FOLFOX and FOLFIRI use in stage IV colon cancer: Analysis of SEER-medicare data. Clin. Color. Cancer.

[16] Xu W., Kuang M., Gong Y., Cao C., Chen J., Tang C. (2016). Survival benefit and safety of the combinations of FOLFOXIRI±bevacizumab versus the combinations of FOLFIRI±bevacizumab as first-line treatment for unresectable metastatic colorectal cancer: A meta-analysis. OncoTargets Ther..

[17] Gmeiner W.H. (2020). Chemistry of Fluorinated Pyrimidines in the Era of Personalized Medicine. Molecules.

[18] Entezar-Almahdi E., Mohammadi-Samani S., Tayebi L., Farjadian F. (2020). Recent advances in designing 5-fluorouracil delivery systems: A stepping stone in the safe treatment of colorectal cancer. Int. J. Nanomed..

[19] Morawska K., Goirand F., Marceau L., Devaux M., Cueff A., Bertaut A., Vincent J., Bengrine-Lefevre L., Ghiringhelli F., Schmitt A. (2018). 5-FU therapeutic drug monitoring as a valuable option to reduce toxicity in patients with gastrointestinal cancer. Oncotarget.

[20] Miura K., Kinouchi M., Ishida K., Fujibuchi W., Naitoh T., Ogawa H., Ando T., Yazaki N., Watanabe K., Haneda S. (2010). 5-fu metabolism in cancer and orally-administrable 5-fu drugs. Cancers.

[21] Gmeiner W.H., Okechukwu C.C. (2023). Review of 5-FU resistance mechanisms in colorectal cancer: Clinical significance of attenuated on-target effects. Cancer Drug Resist..

[22] Diasio R.B., Harris B.E. (1989). Clinical pharmacology of 5-fluorouracil. Clin. Pharmacokinet..

[23] Amidon S., Brown J.E., Dave V.S. (2015). Colon-targeted oral drug delivery systems: Design trends and approaches. AAPS PharmSciTech.

[24] Belali N., Wathoni N., Muchtaridi M. (2019). Advances in orally targeted drug delivery to colon. J. Adv. Pharm. Technol. Res..

[25] Wang N., Chen L., Huang W., Gao Z., Jin M. (2024). Current Advances of Nanomaterial-Based Oral Drug Delivery for Colorectal Cancer Treatment. Nanomaterials.

[26] Wang Z., Yang L. (2022). Broad-spectrum prodrugs with anti-SARS-CoV-2 activities: Strategies, benefits, and challenges. J. Med. Virol..

[27] Xu X., Li Z., Yao X., Sun N., Chang J. (2023). Advanced prodrug strategies in nucleoside analogues targeting the treatment of gastrointestinal malignancies. Front. Cell Dev. Biol..

[28] Miwa M., Ura M., Nishida M., Sawada N., Ishikawa T., Mori K., Shimma N., Umeda I., Ishitsuka H. (1998). Design of a novel oral fluoropyrimidine carbamate, capecitabine, which generates 5-fluorouracil selectively in tumours by enzymes concentrated in human liver and cancer tissue. Eur. J. Cancer.

[29] Derissen E.J., Jacobs B.A., Huitema A.D., Rosing H., Schellens J.H., Beijnen J.H. (2016). Exploring the intracellular pharmacokinetics of the 5-fluorouracil nucleotides during capecitabine treatment. Br. J. Clin. Pharmacol..

[30] Giller S., Zhuk R., MIu L. (1967). Analogs of pyrimidine nucleosides. I. N1-(alpha-furanidyl) derivatives of natural pyrimidine bases and their antimetabolities. Dokl. Akad. Nauk SSSR.

[31] Lembersky B.C., Wieand H.S., Petrelli N.J., O’Connell M.J., Colangelo L.H., Smith R.E., Seay T.E., Giguere J.K., Marshall M.E., Jacobs A.D. (2006). Oral uracil and tegafur plus leucovorin compared with intravenous fluorouracil and leucovorin in stage II and III carcinoma of the colon: Results from National Surgical Adjuvant Breast and Bowel Project Protocol C-06. J. Clin. Oncol..

[32] Yoshisue K., Hironaga K., Yamaguchi S., Yamamoto A., Nagayama S., Kawaguchi Y. (2000). Reduction of 5-fluorouracil (5-FU) gastrointestinal (GI) toxicity resulting from the protection of thymidylate synthase (TS) in GI tissue by repeated simultaneous administration of potassium oxonate (Oxo) in rats. Cancer Chemother. Pharmacol..

[33] Cook A., Holman M., Kramer M., Trown P. (1979). Fluorinated pyrimidine nucleosides. 3. Synthesis and antitumor activity of a series of 5′-deoxy-5-fluoropyrimidine nucleosides. J. Med. Chem..

[34] Ogata Y., Sasatomi T., Mori S., Matono K., Ishibashi N., Akagi Y., Fukushima T., Murakami H., Ushijima M., Shirouzu K. (2007). Significance of thymidine phosphorylase in metronomic chemotherapy using CPT-11 and doxifluridine for advanced colorectal carcinoma. Anticancer. Res..

[35] Ciaffaglione V., Modica M.N., Pittala V., Romeo G., Salerno L., Intagliata S. (2021). Mutual Prodrugs of 5-Fluorouracil: From a Classic Chemotherapeutic Agent to Novel Potential Anticancer Drugs. ChemMedChem.

[36] Desreumaux P., Ghosh S. (2006). Review article: Mode of action and delivery of 5-aminosalicylic acid—New evidence. Aliment. Pharmacol. Ther..

[37] Jiang Y., Li X., Li X., Hou J., Ding Y., Zhang J., Xu W., Zhang Y. (2016). Discovery of Multi-target Anticancer Agents Based on HDAC Inhibitor MS-275 and 5-FU. Med. Chem..

[38] Guan X.W., Xu X.H., Feng S.L., Tang Z.B., Chen S.W., Hui L. (2016). Synthesis of hybrid 4-deoxypodophyllotoxin-5-fluorouracil compounds that inhibit cellular migration and induce cell cycle arrest. Bioorg. Med. Chem. Lett..

[39] Zhang R., Song X.Q., Liu R.P., Ma Z.Y., Xu J.Y. (2019). Fuplatin: An Efficient and Low-Toxic Dual-Prodrug. J. Med. Chem..

[40] Thysiadis S., Katsamakas S., Dalezis P., Chatzisideri T., Trafalis D., Sarli V. (2017). Novel c(RGDyK)-based conjugates of POPAM and 5-fluorouracil for integrin-targeted cancer therapy. Future Med. Chem..

[41] Ito T., Tanabe K., Yamada H., Hatta H., Nishimoto S. (2008). Radiation- and photo-induced activation of 5-fluorouracil prodrugs as a strategy for the selective treatment of solid tumors. Molecules.

[42] Han H.H., Wang H.M., Jangili P., Li M., Wu L., Zang Y., Sedgwick A.C., Li J., He X.P., James T.D. (2023). The design of small-molecule prodrugs and activatable phototherapeutics for cancer therapy. Chem. Soc. Rev..

[43] Liu W., Liu H., Peng X., Zhou G., Liu D., Li S., Zhang J., Wang S. (2018). Hypoxia-Activated Anticancer Prodrug for Bioimaging, Tracking Drug Release, and Anticancer Application. Bioconjug. Chem..

[44] Weckbecker G. (1991). Biochemical pharmacology and analysis of fluoropyrimidines alone and in combination with modulators. Pharmacol. Ther..

[45] Gmeiner W.H. (2005). Novel chemical strategies for thymidylate synthase inhibition. Curr. Med. Chem..

[46] Bre J., Dickson A.L., Read O.J., Zhang Y., McKissock F.G., Mullen P., Tang P., Zickuhr G.M., Czekster C.M., Harrison D.J. (2023). The novel anti-cancer fluoropyrimidine NUC-3373 is a potent inhibitor of thymidylate synthase and an effective DNA-damaging agent. Cancer Chemother. Pharmacol..

[47] Mehellou Y., Rattan H.S., Balzarini J. (2018). The ProTide Prodrug Technology: From the Concept to the Clinic. J. Med. Chem..

[48] Sun Y.W., Chen K.M., Kwon C.H. (2006). Sulfonyl-containing nucleoside phosphotriesters and phosphoramidates as novel anticancer prodrugs of 5-fluoro-2′-deoxyuridine 5′-monophosphate (FdUMP). Mol. Pharm..

[49] Gogoi H., Mansouri S., Jin L. (2020). The age of cyclic dinucleotide vaccine adjuvants. Vaccines.

[50] Xie Z., Yang Y., Wang Z., Ma D., Xi Z. (2023). Dithioethanol (DTE)-Conjugated Deoxyribose Cyclic Dinucleotide Prodrugs (DTE-dCDNs) as STING Agonist. Int. J. Mol. Sci..

[51] Chuang J.C., Warner S.L., Vollmer D., Vankayalapati H., Redkar S., Bearss D.J., Qiu X., Yoo C.B., Jones P.A. (2010). S110, a 5-Aza-2′-deoxycytidine–containing dinucleotide, is an effective DNA methylation inhibitor in vivo and can reduce tumor growth. Mol. Cancer Ther..

[52] Liu J., Skradis A., Kolar C., Kolath J., Anderson J., Lawson T., Talmadge J., Gmeiner W.H. (1999). Increased cytotoxicity and decreased in vivo toxicity of FdUMP [10] relative to 5-FU. Nucleosides Nucleotides.

[53] Liao Z.-Y., Sordet O., Zhang H.-L., Kohlhagen G., Antony S., Gmeiner W.H., Pommier Y. (2005). A novel polypyrimidine antitumor agent FdUMP [10] induces thymineless death with topoisomerase I-DNA complexes. Cancer Res..

[54] Mani C., Pai S., Papke C.M., Palle K., Gmeiner W.H. (2018). Thymineless death by the fluoropyrimidine polymer F10 involves replication fork collapse and is enhanced by Chk1 inhibition. Neoplasia.

[55] Liu J., Kolar C., Lawson T.A., Gmeiner W.H. (2001). Targeted drug delivery to chemoresistant cells: Folic acid derivatization of FdUMP [10] enhances cytotoxicity toward 5-FU-resistant human colorectal tumor cells. J. Org. Chem..

[56] Haber A.O., Jain A., Mani C., Nevler A., Agostini L.C., Golan T., Palle K., Yeo C.J., Gmeiner W.H., Brody J.R. (2021). AraC-FdUMP[10] is a next-generation fluoropyrimidine with potent antitumor activity in PDAC and synergy with PARG inhibition. Mol. Cancer Res..

[57] Avino A., Clua A., Bleda M.J., Eritja R., Fabrega C. (2021). Evaluation of Floxuridine Oligonucleotide Conjugates Carrying Potential Enhancers of Cellular Uptake. Int. J. Mol. Sci..

[58] Ghosh S., Salsbury F.R., Horita D.A., Gmeiner W.H. (2011). Zn^2+^ selectively stabilizes FdU-substituted DNA through a unique major groove binding motif. Nucleic Acids Res..

[59] Ghosh S., Salsbury F.R., Horita D.A., Gmeiner W.H. (2013). Cooperative stabilization of Zn(2+):DNA complexes through netropsin binding in the minor groove of FdU-substituted DNA. J. Biomol. Struct. Dyn..

[60] Ghosh S., Mallick S., Das U., Verma A., Pal U., Chatterjee S., Nandy A., Saha K.D., Maiti N.C., Baishya B. (2018). Curcumin stably interacts with DNA hairpin through minor groove binding and demonstrates enhanced cytotoxicity in combination with FdU nucleotides. Biochim. Biophys. Acta Gen. Subj..

[61] Kruspe S., Hahn U. (2014). An Aptamer Intrinsically Comprising 5-Fluoro-2′-deoxyuridine for Targeted Chemotherapy. Angew. Chem. Int. Ed..

[62] Rothemund P.W. (2006). Folding DNA to create nanoscale shapes and patterns. Nature.

[63] Jiang Q., Song C., Nangreave J., Liu X., Lin L., Qiu D., Wang Z.-G., Zou G., Liang X., Yan H. (2012). DNA origami as a carrier for circumvention of drug resistance. J. Am. Chem. Soc..

[64] Kumar M., Jha A., Mishra B. (2024). DNA-Based Nanostructured Platforms as Drug Delivery Systems. Chem Bio Eng..

[65] Jorge A.F., Aviñó A., Pais A.A., Eritja R., Fàbrega C. (2018). DNA-based nanoscaffolds as vehicles for 5-fluoro-2′-deoxyuridine oligomers in colorectal cancer therapy. Nanoscale.

[66] Wang Z., Yang L. (2024). Natural-product-based, carrier-free, noncovalent nanoparticles for tumor chemo-photodynamic combination therapy. Pharmacol. Res..

[67] Giri P.M., Banerjee A., Layek B. (2023). A Recent Review on Cancer Nanomedicine. Cancers.

[68] Bauri S., Tripathi S., Choudhury A.M., Mandal S.S., Raj H., Maiti P. (2023). Nanomaterials as Theranostic Agents for Cancer Therapy. ACS Appl. Nano Mater..

[69] Zhao P., Wang Y., Wu A., Rao Y., Huang Y. (2018). Roles of albumin-binding proteins in cancer progression and biomimetic targeted drug delivery. ChemBioChem.

[70] Kalındemirtaş F.D., Kariper İ.A., Sert E., Okşak N., Kuruca S.E. (2022). The evaluation of anticancer activity by synthesizing 5FU loaded albumin nanoparticles by exposure to UV light. Toxicol. In Vitro.

[71] Jin C., Zhang H., Zou J., Liu Y., Zhang L., Li F., Wang R., Xuan W., Ye M., Tan W. (2018). Floxuridine homomeric oligonucleotides “hitchhike” with albumin in situ for cancer chemotherapy. Angew. Chem. Int. Ed..

[72] She X.D., Chen L.J., Li C.P., He C.Z., He L., Kong L.X. (2015). Functionalization of Hollow Mesoporous Silica Nanoparticles for Improved 5-FU Loading. J. Nanomater..

[73] Moodley T., Singh M. (2019). Polymeric Mesoporous Silica Nanoparticles for Enhanced Delivery of 5-Fluorouracil In Vitro. Pharmaceutics.

[74] Farjadian F., Moghadam M., Monfared M., Mohammadi-Samani S. (2022). Mesoporous silica nanostructure modified with azo gatekeepers for colon targeted delivery of 5-fluorouracil. Aiche J..

[75] Chen L., She X., Wang T., He L., Shigdar S., Duan W., Kong L. (2015). Overcoming acquired drug resistance in colorectal cancer cells by targeted delivery of 5-FU with EGF grafted hollow mesoporous silica nanoparticles. Nanoscale.

[76] Pan G., Jia T.T., Huang Q.X., Qiu Y.Y., Xu J., Yin P.H., Liu T. (2017). Mesoporous silica nanoparticles (MSNs)-based organic/inorganic hybrid nanocarriers loading 5-Fluorouracil for the treatment of colon cancer with improved anticancer efficacy. Colloids Surf. B Biointerfaces.

[77] Zhao S., Sun S., Jiang K., Wang Y., Liu Y., Wu S., Li Z., Shu Q., Lin H. (2019). In Situ Synthesis of Fluorescent Mesoporous Silica-Carbon Dot Nanohybrids Featuring Folate Receptor-Overexpressing Cancer Cell Targeting and Drug Delivery. Nanomicro Lett..

[78] Ibrahim B., Mady O.Y., Tambuwala M.M., Haggag Y.A. (2022). pH-sensitive nanoparticles containing 5-fluorouracil and leucovorin as an improved anti-cancer option for colon cancer. Nanomedicine.

[79] Mattos A.C., Altmeyer C., Tominaga T.T., Khalil N.M., Mainardes R.M. (2016). Polymeric nanoparticles for oral delivery of 5-fluorouracil: Formulation optimization, cytotoxicity assay and pre-clinical pharmacokinetics study. Eur. J. Pharm. Sci..

[80] Wu P., Zhou Q., Zhu H., Zhuang Y., Bao J. (2020). Enhanced antitumor efficacy in colon cancer using EGF functionalized PLGA nanoparticles loaded with 5-Fluorouracil and perfluorocarbon. BMC Cancer.

[81] Wang Y., Li P., Chen L., Gao W., Zeng F., Kong L.X. (2015). Targeted delivery of 5-fluorouracil to HT-29 cells using high efficient folic acid-conjugated nanoparticles. Drug Deliv..

[82] Pangeni R., Choi S.W., Jeon O.-C., Byun Y., Park J.W. (2016). Multiple nanoemulsion system for an oral combinational delivery of oxaliplatin and 5-fluorouracil: Preparation and in vivo evaluation. Int. J. Nanomed..

[83] Jain P., Patel K., Jangid A.K., Guleria A., Patel S., Pooja D., Kulhari H. (2020). Modulating the delivery of 5-fluorouracil to human colon cancer cells using multifunctional arginine-coated manganese oxide nanocuboids with MRI properties. ACS Appl. Bio Mater..

[84] Handali S., Moghimipour E., Rezaei M., Ramezani Z., Kouchak M., Amini M., Angali K.A., Saremy S., Dorkoosh F.A. (2018). A novel 5-Fluorouracil targeted delivery to colon cancer using folic acid conjugated liposomes. Biomed. Pharmacother..

[85] Das U., Bhuniya A., Roy A.K., Gmeiner W.H., Ghosh S. (2020). Hairpin Oligonucleotide Can Functionalize Gold Nanorods for in Vivo Application Delivering Cytotoxic Nucleotides and Curcumin: A Comprehensive Study in Combination with Near-Infrared Laser. ACS Omega.

[86] Das U., Sahoo A., Haldar S., Bhattacharya S., Mandal S.S., Gmeiner W.H., Ghosh S. (2018). Secondary Structure-Dependent Physicochemical Interaction of Oligonucleotides with Gold Nanorod and Photothermal Effect for Future Applications: A New Insight. ACS Omega.

[87] Liu W., Li X., Wong Y.-S., Zheng W., Zhang Y., Cao W., Chen T. (2012). Selenium nanoparticles as a carrier of 5-fluorouracil to achieve anticancer synergism. ACS Nano.

[88] Xu J., Zhang G., Luo X., Wang D., Zhou W., Zhang Y., Zhang W., Chen J., Meng Q., Chen E. (2021). Co-delivery of 5-fluorouracil and miRNA-34a mimics by host-guest self-assembly nanocarriers for efficacious targeted therapy in colorectal cancer patient-derived tumor xenografts. Theranostics.

[89] Abo-Zaid O.A.R., Moawed F.S.M., Barakat W.E.M., Ghobashy M.M., Ahmed E.S.A. (2023). Antitumor activity of 5-fluorouracil polymeric nanogel synthesized by gamma radiation on a rat model of colon carcinoma: A proposed mechanism. Discov. Oncol..

[90] Sadeghi-Abandansari H., Pakian S., Nabid M.-R., Ebrahimi M., Rezalotfi A. (2021). Local co-delivery of 5-fluorouracil and curcumin using Schiff’s base cross-linked injectable hydrogels for colorectal cancer combination therapy. Eur. Polym. J..

[91] Xu L., Wang X., Liu Y., Yang G., Falconer R.J., Zhao C.-X. (2022). Lipid nanoparticles for drug delivery. Adv. NanoBiomed Res..

[92] Smith T., Affram K., Nottingham E.L., Han B., Amissah F., Krishnan S., Trevino J., Agyare E. (2020). Application of smart solid lipid nanoparticles to enhance the efficacy of 5-fluorouracil in the treatment of colorectal cancer. Sci. Rep..

[93] Wang X.-Y., Zheng Z.-X., Sun Y., Bai Y.-H., Shi Y.-F., Zhou L.-X., Yao Y.-F., Wu A.-W., Cao D.-F. (2019). Significance of HER2 protein expression and HER2 gene amplification in colorectal adenocarcinomas. World J. Gastrointest. Oncol..

[94] Kumah E.A., Fopa R.D., Harati S., Boadu P., Zohoori F.V., Pak T. (2023). Human and environmental impacts of nanoparticles: A scoping review of the current literature. BMC Public Health.

[95] Brand W., Noorlander C.W., Giannakou C., De Jong W.H., Kooi M.W., Park M.V., Vandebriel R.J., Bosselaers I.E., Scholl J.H., Geertsma R.E. (2017). Nanomedicinal products: A survey on specific toxicity and side effects. Int. J. Nanomed..

[96] Yang C., Merlin D. (2023). Challenges to safe nanomedicine treatment. Nanomaterials.

[97] Wolfram J., Zhu M., Yang Y., Shen J., Gentile E., Paolino D., Fresta M., Nie G., Chen C., Shen H. (2015). Safety of nanoparticles in medicine. Curr. Drug Targets.

[98] Anselmo A.C., Mitragotri S. (2019). Nanoparticles in the clinic: An update. Bioeng. Transl. Med..

[99] Gmeiner W.H. (2024). Recent Advances in Therapeutic Strategies to Improve Colorectal Cancer Treatment. Cancers.

[100] Wilson P.M., Danenberg P.V., Johnston P.G., Lenz H.J., Ladner R.D. (2014). Standing the test of time: Targeting thymidylate biosynthesis in cancer therapy. Nat. Rev. Clin. Oncol..

